# Di-*μ*_2_-acetato-1:2*κ* ^2^ *O*:*O*′;2:3*κ* ^2^ *O*:*O*′-bis­{*μ*_2_-4,4′-di­chloro-2,2′-[2,2-di­methyl­propane-1,3-diylbis(nitrilo­methanylyl­idene)]diphenolato}-1:2*κ* ^6^ *O*,*N*,*N*′,*O*′:*O*,*O*′;2:3*κ* ^6^ *O*,*O*′:*O*,*N*,*N*′,*O*′-tri­cadmium

**DOI:** 10.1107/S1600536813029413

**Published:** 2013-11-06

**Authors:** Koji Kubono, Keita Tani, Kunihiko Yokoi, Teruo Shinmyozu, Kenta Goto

**Affiliations:** a Division of Natural Sciences, Osaka Kyoiku University, Kashiwara, Osaka 582-8582, Japan; b Institute for Materials Chemistry and Engineering, Kyushu University, Hakozaki 6-10-1, Higashi-ku, Fukuoka 812-8581, Japan

## Abstract

In the title linear homo-trinuclear complex, [Cd_3_(C_19_H_18_Cl_2_N_2_O_2_)_2_(C_2_H_3_O_2_)_2_], the central Cd^II^ atom is located on a centre of inversion and has a distorted octa­hedral coordination geometry formed by four O atoms from two bidentate/tetra­dentate Schiff base ligands and two O atoms from two bridging acetate ligands. The coordination geometry of the terminal Cd^II^ atom is square-pyramidal with the tetra­dentate part of the ligand in the basal plane and one O atom from an acetate ligand occupying the apical site. The six-membered CdN_2_C_3_ ring adopts a chair conformation. The acetate-bridged Cd⋯Cd distance is 3.3071 (2) Å. The crystal structure is stabilized by C—H⋯O hydrogen bonds, which form *C*(7) chain motifs and give rise to a two-dimensional supra­molecular network structure lying parallel to the *ab* plane.

## Related literature

For metalloligands, see: Du *et al.* (2012 ▶); Carlucci *et al.* (2011 ▶); Das *et al.* (2011 ▶). Metal complexes with the Schiff base ligand, bis­(salicyl­idene)propane-1,3-di­amine can be metalloligands, forming linear homo- or hetero-trinuclear complexes with divalent metal salts, see: Atakol, Arıcı *et al.* (1999 ▶), Das *et al.* (2013 ▶); Fukuhara *et al.* (1990 ▶). For related structures, see: Atakol, Aksu *et al.* (1999 ▶); Kubono *et al.* (2012 ▶); Xue *et al.* (2012 ▶). For analysis of ring conformations, see: Cremer & Pople (1975 ▶). For hydrogen-bond motifs, see: Bernstein *et al.* (1995 ▶).
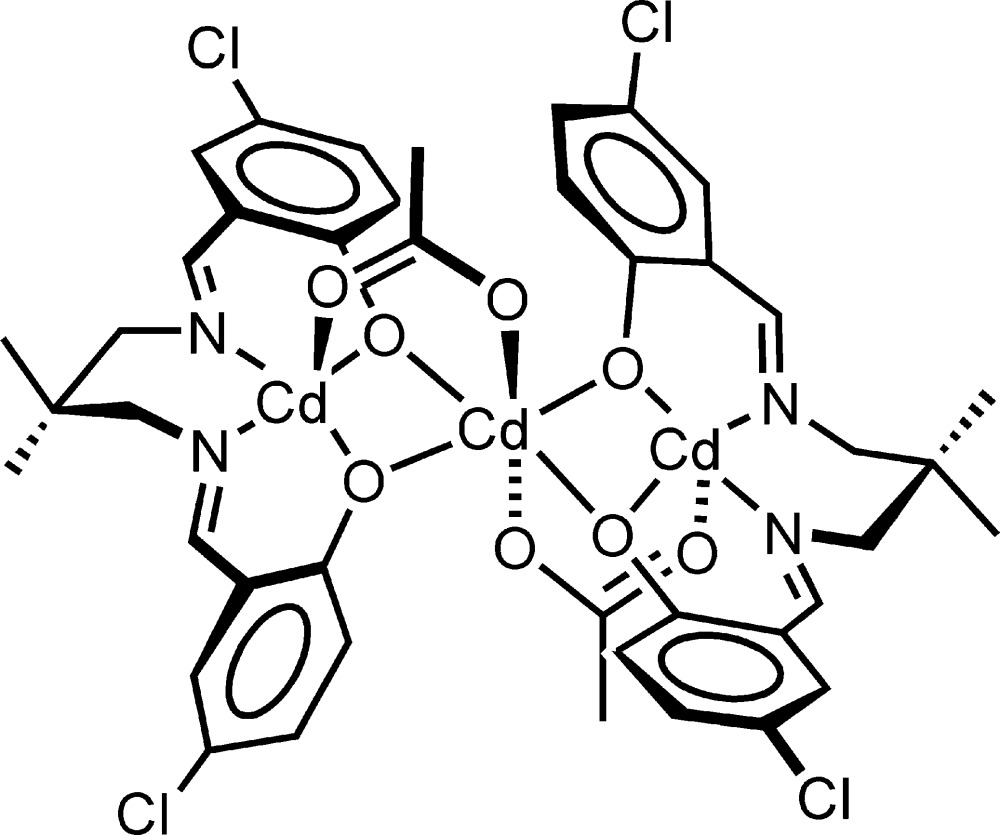


## Experimental

### 

#### Crystal data

[Cd_3_(C_19_H_18_Cl_2_N_2_O_2_)_2_(C_2_H_3_O_2_)_2_]
*M* *_r_* = 1209.83
Orthorhombic, 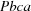

*a* = 19.3078 (15) Å
*b* = 11.2651 (8) Å
*c* = 20.535 (3) Å
*V* = 4466.5 (8) Å^3^
*Z* = 4
Mo *K*α radiation
μ = 1.71 mm^−1^
*T* = 123 K
0.21 × 0.16 × 0.11 mm

#### Data collection

Rigaku RAPID-HR diffractometer
Absorption correction: multi-scan (*ABSCOR*; Higashi, 1995 ▶) *T*_min_ = 0.727, *T*_max_ = 0.828
70676 measured reflections
5107 independent reflections
4990 reflections with *I* > 2σ(*I*)
*R*_int_ = 0.025

#### Refinement

*R*[*F* ^2^ > 2σ(*F* ^2^)] = 0.019
*wR*(*F* ^2^) = 0.058
*S* = 1.00
5107 reflections
280 parameters
H-atom parameters constrained
Δρ_max_ = 0.43 e Å^−3^
Δρ_min_ = −0.84 e Å^−3^

### 

Data collection: *RAPID-AUTO* (Rigaku, 2006 ▶); cell refinement: *RAPID-AUTO*; data reduction: *RAPID-AUTO*; program(s) used to solve structure: *SIR92* (Altomare, *et al.*, 1993 ▶); program(s) used to refine structure: *SHELXL97* (Sheldrick, 2008 ▶); molecular graphics: *PLATON* (Spek, 2009) ▶; software used to prepare material for publication: *CrystalStructure* (Rigaku, 2010 ▶).

## Supplementary Material

Crystal structure: contains datablock(s) General, I. DOI: 10.1107/S1600536813029413/cq2008sup1.cif

Structure factors: contains datablock(s) I. DOI: 10.1107/S1600536813029413/cq2008Isup2.hkl

Additional supplementary materials:  crystallographic information; 3D view; checkCIF report

## Figures and Tables

**Table 1:** Hydrogen-bond geometry (Å, °)

*D*—H⋯*A*	*D*—H	H⋯*A*	*D*⋯*A*	*D*—H⋯*A*
C15—H15⋯O3^i^	0.95	2.54	3.248 (2)	131

Symmetry code: (i) 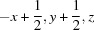.

**Fig. 1.**
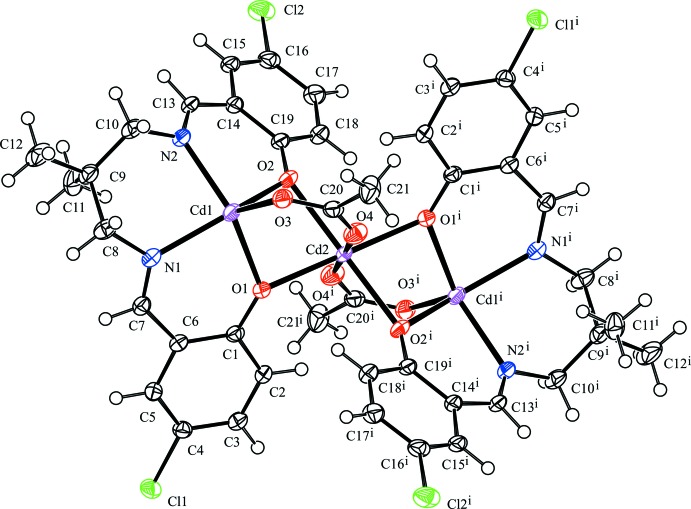
The title compound with displacement ellipsoids drawn at the 50% probability level. H atoms are represented by spheres of arbitrary radius. [symmetry code: (i) -x + 1, -y, -z + 1.]

**Fig. 2.**
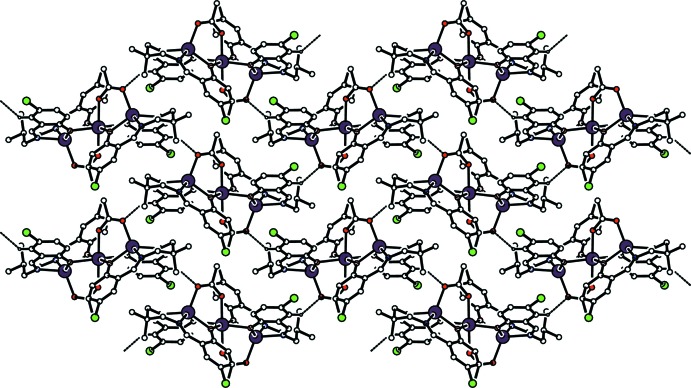
Two-dimensional supramolecular network structure of the title compound. The intermolecular C—H···O hydrogen bonds are shown as dashed lines.

## supplementary crystallographic information

### 1. Comment

Metalloligands are metal-complex molecules which can act as ligands by reacting with other metal ions to form, for example, polynuclear complexes, coordination polymers and metal-organic frameworks. Recently metalloligands have received much attention, because of their functional properties and many potential applications, such as luminescence, catalysis, magnetism, gas storage, ion recognition, see: Du *et al.* (2012), Carlucci *et al.* (2011) and Das *et al.* (2011). The metal complexes with Schiff base ligand, bis(salicylidene)propane-1,3-diamine can be metalloligands, forming linear homo- or hetero-triuclear complexes with divalent metal salts, see: Atakol, Arıcı *et al.* (1999), Das *et al.* (2013) and Fukuhara *et al.* (1990). We have recently studied the structure of a homo-trinuclear Cu^II^ complex with tetradentate bis-chlorosalicylidene, 4,4'-dichloro-2,2'-[2,2-dimethylpropane-1,3- diylbis(nitrilomethanylylidene)]diphenol and copper acetate units as the building blocks. (Kubono *et al.*, 2012). It can be considered that the compound is a 1:2 metal-complex between a copper(II) ion and a Cu^II^ mononuclear complex, which acts as a metalloligand. Subsequently, we have tried to synthesize further trinuclear complexes with the same ligand and other metal ions. Herein, the structure of the title cadmium-based trinuclear complex, containing the tetradentate Schiff base ligand and cadmium acetate units, is reported.

The central Cd^II^ atom, Cd2, is located on a centre of inversion and has a distorted octahedral coordination enviroment, formed by four oxygen atoms from two tetradentate Schiff base ligands in the equatorial plane and an oxygen atom from each of the two bridging acetate ligands in the axial positions. The terminal Cd^II^ atom, Cd1, has a distorted square-pyramidal configuration with atoms in the basal plane comprising two phenolate O and two imine N atoms from the tetradentate ligand. The apical site is occupied by one O atom from an acetate bridging ligand. Cd1 is located at 0.77466 (10) Å above the mean basal plane (N1/N2/O1/O2) of the square-based pyramid. The six-membered Cd1/N1/C8/C9/C10/N2 ring adopts a chair conformation with puckering parameters (Cremer & Pople, 1975): Q = 0.6280 (15) Å, θ = 3.29 (14)° and φ = 251 (2)°. The bond lengths and angles involving Cd^II^ atoms are comparable to those observed in related linear homo-trinuclear Cd^II^ complexes (Atakol, Aksu *et al.*, 1999; Xue *et al.*, 2012). The dihedral angle between the benzene rings (C1–C6 and C14–C19) is 71.88 (7)°, a value comparable with that found in the related trinuclear Cu^II^ complex (Kubono *et al.*, 2012). The Cd1···Cd2 distance is 3.3071 (2) Å, similar to that found in related structures (Atakol, Aksu *et al.*, 1999; Xue *et al.*, 2012). In the crystal structure of the title complex, there is an intermolecular C15—H15···O3^i^ hydrogen bond [symmetry code: (i) -*x* + 1/2, *y* + 1/2, *z*; Table 1], forming a *C*(7) chain motif (Bernstein *et al.*, 1995). C15—H in the benzene ring at (*x*, *y*, *z*) acts as hydrogen bond donor to atom O3 from an acetate at (-*x* + 1/2, *y* + 1/2, *z*), so forming a *C*(7) chain running parallel to the *b*-axis and generated by the *b*-glide plane at *x* = 1/4. The crystal structure is stabilised by intermolecular C—H···O hydrogen bonds, which form a two-dimensional supramolecular network structure parallel to the *ab* plane with an *R*_4_^4^(34) graph-set ring motif (Fig. 2).

### 2. Experimental

The Schiff base ligand, 4'-dichloro-2,2'-[2,2-dimethylpropane-1,3- diylbis(nitrilomethanylylidene)]diphenol, (0.40 mmol) was dissolved in 20 ml hot methanol. A solution of cadmium acetate dihydrate (0.60 mmol) in 20 ml hot methanol was then added to the ligand solution. The mixture was stirred for 20 min at 340 K. After a few weeks at room temperature, colorless crystals of the title complex were obtained. Yield 48%. Analysis calculated for C_42_H_42_Cd_3_Cl_4_N_4_O_8_: C 41.70, H 3.50, N 4.63%; found: C 41.50, H 3.44, N 4.33%.

### 3. Refinement

All H atoms bound to carbon were placed at idealized positions and refined using a riding model, with C—H = 0.95–0.99 Å and *U*_iso_(H) = 1.2*U*_eq_(C).

### Crystal data

**Table:**

[Cd_3_(C_19_H_18_Cl_2_N_2_O_2_)_2_(C_2_H_3_O_2_)_2_]	*F*(000) = 2392.00
*M**_r_* = 1209.83	*D*_x_ = 1.799 Mg m^−^^3^
Orthorhombic, *P**b**c**a*	Mo *K*α radiation, λ = 0.71075 Å
Hall symbol: -P 2ac 2ab	Cell parameters from 65865 reflections
*a* = 19.3078 (15) Å	θ = 3.2–27.5°
*b* = 11.2651 (8) Å	µ = 1.71 mm^−^^1^
*c* = 20.535 (3) Å	*T* = 123 K
*V* = 4466.5 (8) Å^3^	Prism, colorless
*Z* = 4	0.21 × 0.16 × 0.11 mm

### Data collection

**Table:**

Rigaku RAPID-HR diffractometer	4990 reflections with *F*^2^ > 2.0σ(*F*^2^)
Detector resolution: 10.00 pixels mm^-1^	*R*_int_ = 0.025
ω scans	θ_max_ = 27.5°
Absorption correction: multi-scan (*ABSCOR*; Higashi, 1995)	*h* = −24→24
*T*_min_ = 0.727, *T*_max_ = 0.828	*k* = −14→14
70676 measured reflections	*l* = −26→26
5107 independent reflections	

### Refinement

**Table:**

Refinement on *F*^2^	Secondary atom site location: difference Fourier map
*R*[*F*^2^ > 2σ(*F*^2^)] = 0.019	Hydrogen site location: inferred from neighbouring sites
*wR*(*F*^2^) = 0.058	H-atom parameters constrained
*S* = 1.00	*w* = 1/[σ^2^(*F*_o_^2^) + (0.043*P*)^2^ + 1.9282*P*] where *P* = (*F*_o_^2^ + 2*F*_c_^2^)/3
5107 reflections	(Δ/σ)_max_ = 0.002
280 parameters	Δρ_max_ = 0.43 e Å^−^^3^
0 restraints	Δρ_min_ = −0.84 e Å^−^^3^
Primary atom site location: structure-invariant direct methods	

### Special details

**Table:**

Geometry. All e.s.d.'s (except the e.s.d. in the dihedral angle between two l.s. planes) are estimated using the full covariance matrix. The cell e.s.d.'s are taken into account individually in the estimation of e.s.d.'s in distances, angles and torsion angles; correlations between e.s.d.'s in cell parameters are only used when they are defined by crystal symmetry. An approximate (isotropic) treatment of cell e.s.d.'s is used for estimating e.s.d.'s involving l.s. planes.
Refinement. Refinement was performed using all reflections. The weighted *R*-factor (*wR*) and goodness of fit (*S*) are based on *F*^2^. *R*-factor (gt) are based on *F*. The threshold expression of *F*^2^ > 2.0 σ(*F*^2^) is used only for calculating *R*-factor (gt).

### Fractional atomic coordinates and isotropic or equivalent isotropic displacement parameters (Å^2^)

**Table:**

	*x*	*y*	*z*	*U*_iso_*/*U*_eq_	
Cd1	0.362773 (5)	0.859740 (9)	0.441968 (5)	0.02095 (5)	
Cd2	0.5000	1.0000	0.5000	0.01883 (5)	
Cl1	0.51482 (2)	1.15865 (3)	0.144318 (18)	0.02734 (8)	
Cl2	0.20809 (2)	1.33156 (4)	0.66126 (2)	0.03710 (10)	
O1	0.45450 (5)	0.95298 (10)	0.40088 (5)	0.0225 (2)	
O2	0.38433 (5)	0.99130 (10)	0.52101 (6)	0.0257 (3)	
O3	0.40248 (6)	0.70129 (10)	0.48903 (5)	0.0250 (3)	
O4	0.49801 (6)	0.79948 (11)	0.51990 (7)	0.0299 (3)	
N1	0.32621 (6)	0.85897 (10)	0.33787 (6)	0.0212 (3)	
N2	0.25153 (6)	0.89528 (12)	0.46924 (6)	0.0251 (3)	
C1	0.46601 (7)	0.99631 (12)	0.34260 (7)	0.0194 (3)	
C2	0.52539 (7)	1.06800 (13)	0.33204 (7)	0.0226 (3)	
C3	0.53978 (8)	1.11685 (13)	0.27195 (7)	0.0237 (3)	
C4	0.49576 (7)	1.09536 (13)	0.21952 (7)	0.0214 (3)	
C5	0.43805 (7)	1.02528 (13)	0.22745 (7)	0.0206 (3)	
C6	0.42155 (7)	0.97504 (12)	0.28852 (7)	0.0192 (3)	
C7	0.35682 (7)	0.90848 (13)	0.28945 (7)	0.0208 (3)	
C8	0.26034 (8)	0.79767 (13)	0.32396 (8)	0.0269 (3)	
C9	0.19671 (8)	0.84468 (14)	0.36087 (8)	0.0258 (3)	
C10	0.19962 (8)	0.82302 (16)	0.43504 (8)	0.0300 (4)	
C11	0.18625 (9)	0.97609 (16)	0.34678 (9)	0.0350 (4)	
C12	0.13541 (9)	0.7716 (3)	0.33557 (11)	0.0470 (6)	
C13	0.22970 (8)	0.97577 (17)	0.50828 (8)	0.0267 (3)	
C14	0.26924 (7)	1.06011 (15)	0.54673 (7)	0.0243 (3)	
C15	0.22917 (8)	1.14259 (15)	0.58208 (8)	0.0276 (4)	
C16	0.25948 (8)	1.22761 (15)	0.62020 (7)	0.0280 (4)	
C17	0.33148 (8)	1.23407 (15)	0.62587 (8)	0.0290 (4)	
C18	0.37173 (8)	1.15406 (15)	0.59164 (8)	0.0264 (4)	
C19	0.34295 (8)	1.06565 (14)	0.55137 (7)	0.0224 (3)	
C20	0.45884 (8)	0.71155 (13)	0.52040 (7)	0.0230 (3)	
C21	0.47888 (10)	0.60752 (18)	0.56294 (10)	0.0379 (4)	
H2	0.5561	1.0827	0.3673	0.0271*	
H3	0.5797	1.1651	0.2663	0.0284*	
H5	0.4088	1.0104	0.1912	0.0247*	
H7	0.3342	0.9006	0.2486	0.0250*	
H8A	0.2661	0.7125	0.3345	0.0322*	
H8B	0.2510	0.8036	0.2767	0.0322*	
H10A	0.1534	0.8400	0.4538	0.0360*	
H10B	0.2099	0.7381	0.4429	0.0360*	
H11A	0.1455	1.0048	0.3705	0.0420*	
H11B	0.1793	0.9876	0.2999	0.0420*	
H11C	0.2272	1.0205	0.3609	0.0420*	
H12A	0.1283	0.7884	0.2892	0.0564*	
H12B	0.0935	0.7928	0.3599	0.0564*	
H12C	0.1452	0.6869	0.3414	0.0564*	
H13	0.1808	0.9810	0.5127	0.0320*	
H15	0.1801	1.1390	0.5794	0.0332*	
H17	0.3524	1.2925	0.6528	0.0348*	
H18	0.4207	1.1589	0.5954	0.0317*	
H21A	0.4760	0.6309	0.6088	0.0454*	
H21B	0.5264	0.5831	0.5527	0.0454*	
H21C	0.4472	0.5412	0.5548	0.0454*	

### Atomic displacement parameters (Å^2^)

**Table:**

	*U*^11^	*U*^22^	*U*^33^	*U*^12^	*U*^13^	*U*^23^
Cd1	0.01340 (7)	0.02433 (7)	0.02512 (8)	0.00059 (3)	0.00053 (3)	0.00617 (4)
Cd2	0.01134 (8)	0.02545 (9)	0.01971 (9)	0.00049 (5)	0.00088 (5)	0.00179 (5)
Cl1	0.02882 (18)	0.03126 (18)	0.02194 (17)	−0.00684 (14)	0.00090 (14)	0.00652 (13)
Cl2	0.0367 (3)	0.0413 (3)	0.0332 (2)	0.01901 (18)	0.01043 (17)	0.00549 (17)
O1	0.0169 (5)	0.0313 (6)	0.0192 (5)	−0.0029 (4)	0.0008 (4)	0.0037 (4)
O2	0.0130 (5)	0.0361 (6)	0.0279 (6)	0.0022 (4)	0.0028 (5)	−0.0027 (5)
O3	0.0213 (5)	0.0280 (6)	0.0257 (5)	0.0032 (5)	0.0015 (4)	0.0065 (4)
O4	0.0257 (6)	0.0267 (6)	0.0372 (7)	0.0003 (4)	0.0035 (5)	0.0063 (5)
N1	0.0161 (6)	0.0207 (6)	0.0269 (7)	−0.0006 (5)	0.0010 (5)	−0.0003 (5)
N2	0.0147 (6)	0.0366 (7)	0.0241 (7)	−0.0010 (6)	0.0008 (5)	0.0066 (5)
C1	0.0156 (7)	0.0212 (7)	0.0214 (7)	0.0021 (5)	0.0024 (6)	0.0014 (5)
C2	0.0158 (7)	0.0299 (7)	0.0219 (7)	−0.0024 (6)	−0.0010 (5)	0.0017 (6)
C3	0.0177 (7)	0.0270 (7)	0.0263 (7)	−0.0040 (6)	0.0016 (6)	0.0014 (6)
C4	0.0210 (7)	0.0232 (7)	0.0200 (7)	0.0003 (5)	0.0026 (5)	0.0029 (6)
C5	0.0187 (7)	0.0228 (6)	0.0203 (7)	0.0007 (6)	−0.0012 (5)	−0.0006 (6)
C6	0.0157 (6)	0.0194 (6)	0.0224 (7)	0.0010 (5)	0.0019 (5)	−0.0007 (5)
C7	0.0176 (7)	0.0208 (7)	0.0241 (7)	0.0008 (5)	−0.0004 (5)	−0.0033 (6)
C8	0.0214 (7)	0.0223 (7)	0.0369 (8)	−0.0065 (6)	0.0044 (6)	−0.0052 (6)
C9	0.0176 (7)	0.0291 (8)	0.0306 (8)	−0.0044 (6)	0.0003 (6)	−0.0040 (6)
C10	0.0177 (7)	0.0377 (9)	0.0346 (9)	−0.0084 (7)	0.0026 (6)	0.0049 (7)
C11	0.0314 (9)	0.0352 (9)	0.0383 (9)	0.0094 (7)	−0.0095 (8)	0.0005 (8)
C12	0.0240 (9)	0.0685 (15)	0.0485 (12)	−0.0192 (9)	0.0050 (8)	−0.0235 (11)
C13	0.0124 (7)	0.0436 (9)	0.0241 (7)	0.0026 (7)	0.0018 (6)	0.0069 (7)
C14	0.0163 (7)	0.0363 (8)	0.0202 (7)	0.0053 (6)	0.0014 (5)	0.0065 (6)
C15	0.0168 (7)	0.0435 (10)	0.0225 (8)	0.0096 (6)	0.0038 (6)	0.0092 (6)
C16	0.0263 (8)	0.0345 (8)	0.0232 (7)	0.0124 (7)	0.0062 (6)	0.0062 (6)
C17	0.0266 (8)	0.0328 (8)	0.0276 (8)	0.0027 (7)	0.0027 (6)	0.0011 (7)
C18	0.0181 (7)	0.0338 (8)	0.0273 (8)	0.0029 (6)	0.0008 (6)	0.0020 (6)
C19	0.0151 (7)	0.0308 (8)	0.0213 (7)	0.0033 (6)	0.0027 (5)	0.0058 (6)
C20	0.0210 (7)	0.0262 (7)	0.0218 (7)	0.0046 (6)	0.0051 (6)	0.0042 (6)
C21	0.0333 (10)	0.0339 (9)	0.0463 (11)	−0.0015 (8)	−0.0108 (8)	0.0164 (8)

### Geometric parameters (Å, º)

**Table:**

Cd1—O1	2.2253 (11)	C9—C12	1.532 (3)
Cd1—O2	2.2370 (12)	C13—C14	1.452 (3)
Cd1—O3	2.1698 (12)	C14—C15	1.410 (3)
Cd1—N1	2.2513 (13)	C14—C19	1.428 (2)
Cd1—N2	2.2555 (12)	C15—C16	1.368 (3)
Cd2—O1	2.2794 (11)	C16—C17	1.397 (3)
Cd2—O1^i^	2.2794 (11)	C17—C18	1.382 (3)
Cd2—O2	2.2767 (10)	C18—C19	1.409 (3)
Cd2—O2^i^	2.2767 (10)	C20—C21	1.512 (3)
Cd2—O4	2.2959 (13)	C2—H2	0.950
Cd2—O4^i^	2.2959 (13)	C3—H3	0.950
Cl1—C4	1.7403 (15)	C5—H5	0.950
Cl2—C16	1.7512 (17)	C7—H7	0.950
O1—C1	1.3115 (18)	C8—H8A	0.990
O2—C19	1.3147 (19)	C8—H8B	0.990
O3—C20	1.2698 (19)	C10—H10A	0.990
O4—C20	1.246 (2)	C10—H10B	0.990
N1—C7	1.2842 (19)	C11—H11A	0.980
N1—C8	1.475 (2)	C11—H11B	0.980
N2—C10	1.470 (2)	C11—H11C	0.980
N2—C13	1.282 (3)	C12—H12A	0.980
C1—C2	1.419 (2)	C12—H12B	0.980
C1—C6	1.424 (2)	C12—H12C	0.980
C2—C3	1.379 (2)	C13—H13	0.950
C3—C4	1.393 (2)	C15—H15	0.950
C4—C5	1.375 (2)	C17—H17	0.950
C5—C6	1.412 (2)	C18—H18	0.950
C6—C7	1.458 (2)	C21—H21A	0.980
C8—C9	1.538 (3)	C21—H21B	0.980
C9—C10	1.544 (3)	C21—H21C	0.980
C9—C11	1.522 (3)		
			
O1—Cd1—O2	79.30 (4)	N2—C13—C14	129.06 (14)
O1—Cd1—O3	106.02 (5)	C13—C14—C15	114.99 (13)
O1—Cd1—N1	83.76 (4)	C13—C14—C19	126.08 (14)
O1—Cd1—N2	140.20 (5)	C15—C14—C19	118.93 (14)
O2—Cd1—O3	98.98 (5)	C14—C15—C16	121.40 (15)
O2—Cd1—N1	138.58 (5)	Cl2—C16—C15	120.08 (12)
O2—Cd1—N2	83.07 (5)	Cl2—C16—C17	119.27 (13)
O3—Cd1—N1	122.04 (4)	C15—C16—C17	120.65 (15)
O3—Cd1—N2	111.83 (5)	C16—C17—C18	118.89 (15)
N1—Cd1—N2	86.44 (5)	C17—C18—C19	122.53 (15)
O1—Cd2—O1^i^	180.00 (6)	O2—C19—C14	123.11 (14)
O1—Cd2—O2	77.36 (4)	O2—C19—C18	119.28 (14)
O1—Cd2—O2^i^	102.64 (4)	C14—C19—C18	117.60 (14)
O1—Cd2—O4	85.63 (5)	O3—C20—O4	126.03 (15)
O1—Cd2—O4^i^	94.37 (5)	O3—C20—C21	116.22 (14)
O1^i^—Cd2—O2	102.64 (4)	O4—C20—C21	117.74 (15)
O1^i^—Cd2—O2^i^	77.36 (4)	C1—C2—H2	119.118
O1^i^—Cd2—O4	94.37 (5)	C3—C2—H2	119.114
O1^i^—Cd2—O4^i^	85.63 (5)	C2—C3—H3	120.024
O2—Cd2—O2^i^	180.00 (6)	C4—C3—H3	120.026
O2—Cd2—O4	84.69 (4)	C4—C5—H5	119.399
O2—Cd2—O4^i^	95.31 (4)	C6—C5—H5	119.406
O2^i^—Cd2—O4	95.31 (4)	N1—C7—H7	115.542
O2^i^—Cd2—O4^i^	84.69 (4)	C6—C7—H7	115.541
O4—Cd2—O4^i^	180.00 (7)	N1—C8—H8A	108.369
Cd1—O1—Cd2	94.46 (4)	N1—C8—H8B	108.371
Cd1—O1—C1	131.04 (9)	C9—C8—H8A	108.384
Cd2—O1—C1	131.54 (9)	C9—C8—H8B	108.387
Cd1—O2—Cd2	94.22 (5)	H8A—C8—H8B	107.442
Cd1—O2—C19	130.77 (10)	N2—C10—H10A	108.721
Cd2—O2—C19	131.19 (10)	N2—C10—H10B	108.717
Cd1—O3—C20	116.99 (10)	C9—C10—H10A	108.725
Cd2—O4—C20	142.52 (11)	C9—C10—H10B	108.728
Cd1—N1—C7	126.10 (10)	H10A—C10—H10B	107.633
Cd1—N1—C8	117.13 (10)	C9—C11—H11A	109.477
C7—N1—C8	116.76 (13)	C9—C11—H11B	109.476
Cd1—N2—C10	115.62 (10)	C9—C11—H11C	109.471
Cd1—N2—C13	126.45 (11)	H11A—C11—H11B	109.472
C10—N2—C13	117.80 (13)	H11A—C11—H11C	109.461
O1—C1—C2	119.22 (13)	H11B—C11—H11C	109.470
O1—C1—C6	123.16 (13)	C9—C12—H12A	109.468
C2—C1—C6	117.62 (13)	C9—C12—H12B	109.476
C1—C2—C3	121.77 (14)	C9—C12—H12C	109.467
C2—C3—C4	119.95 (14)	H12A—C12—H12B	109.466
Cl1—C4—C3	119.06 (11)	H12A—C12—H12C	109.472
Cl1—C4—C5	120.77 (11)	H12B—C12—H12C	109.479
C3—C4—C5	120.17 (14)	N2—C13—H13	115.482
C4—C5—C6	121.20 (13)	C14—C13—H13	115.461
C1—C6—C5	119.28 (13)	C14—C15—H15	119.306
C1—C6—C7	126.39 (13)	C16—C15—H15	119.296
C5—C6—C7	114.28 (13)	C16—C17—H17	120.554
N1—C7—C6	128.92 (14)	C18—C17—H17	120.553
N1—C8—C9	115.61 (13)	C17—C18—H18	118.727
C8—C9—C10	113.76 (13)	C19—C18—H18	118.741
C8—C9—C11	110.32 (13)	C20—C21—H21A	109.462
C8—C9—C12	105.37 (14)	C20—C21—H21B	109.465
C10—C9—C11	110.26 (14)	C20—C21—H21C	109.462
C10—C9—C12	106.13 (14)	H21A—C21—H21B	109.487
C11—C9—C12	110.83 (15)	H21A—C21—H21C	109.485
N2—C10—C9	114.13 (14)	H21B—C21—H21C	109.466
			
O1—Cd1—O2—Cd2	−28.74 (4)	O4—Cd2—O2—C19	142.33 (11)
O1—Cd1—O2—C19	130.57 (11)	O2—Cd2—O4^i^—C20^i^	156.99 (16)
O2—Cd1—O1—Cd2	28.71 (4)	O4^i^—Cd2—O2—Cd1	121.51 (5)
O2—Cd1—O1—C1	−133.05 (10)	O4^i^—Cd2—O2—C19	−37.67 (11)
O1—Cd1—O3—C20	39.81 (8)	O2^i^—Cd2—O4—C20	−156.99 (16)
O3—Cd1—O1—Cd2	−67.66 (5)	O4—Cd2—O2^i^—Cd1^i^	−121.51 (5)
O3—Cd1—O1—C1	130.58 (9)	O4—Cd2—O2^i^—C19^i^	37.67 (11)
O1—Cd1—N1—C7	−4.33 (9)	O2^i^—Cd2—O4^i^—C20^i^	−23.01 (16)
O1—Cd1—N1—C8	176.77 (8)	O4^i^—Cd2—O2^i^—Cd1^i^	58.49 (5)
N1—Cd1—O1—Cd2	170.75 (5)	O4^i^—Cd2—O2^i^—C19^i^	−142.33 (11)
N1—Cd1—O1—C1	8.98 (9)	Cd1—O1—C1—C2	170.40 (7)
O1—Cd1—N2—C10	120.25 (8)	Cd1—O1—C1—C6	−9.61 (19)
O1—Cd1—N2—C13	−55.53 (13)	Cd2—O1—C1—C2	15.04 (18)
N2—Cd1—O1—Cd2	93.98 (7)	Cd2—O1—C1—C6	−164.98 (8)
N2—Cd1—O1—C1	−67.79 (12)	Cd1—O2—C19—C14	12.4 (2)
O2—Cd1—O3—C20	−41.55 (8)	Cd1—O2—C19—C18	−168.95 (8)
O3—Cd1—O2—Cd2	76.00 (5)	Cd2—O2—C19—C14	164.46 (9)
O3—Cd1—O2—C19	−124.68 (10)	Cd2—O2—C19—C18	−16.9 (2)
O2—Cd1—N1—C7	61.70 (12)	Cd1—O3—C20—O4	−9.83 (19)
O2—Cd1—N1—C8	−117.20 (7)	Cd1—O3—C20—C21	169.05 (7)
N1—Cd1—O2—Cd2	−96.32 (6)	Cd2—O4—C20—O3	22.9 (3)
N1—Cd1—O2—C19	62.99 (12)	Cd2—O4—C20—C21	−155.94 (13)
O2—Cd1—N2—C10	−175.73 (9)	Cd1—N1—C7—C6	0.7 (2)
O2—Cd1—N2—C13	8.50 (10)	Cd1—N1—C8—C9	58.78 (13)
N2—Cd1—O2—Cd2	−172.89 (5)	C7—N1—C8—C9	−120.22 (14)
N2—Cd1—O2—C19	−13.58 (10)	C8—N1—C7—C6	179.65 (12)
O3—Cd1—N1—C7	−109.35 (9)	Cd1—N2—C10—C9	−64.13 (14)
O3—Cd1—N1—C8	71.75 (9)	Cd1—N2—C13—C14	−3.0 (3)
N1—Cd1—O3—C20	132.47 (7)	C10—N2—C13—C14	−178.65 (14)
O3—Cd1—N2—C10	−78.79 (8)	C13—N2—C10—C9	112.03 (16)
O3—Cd1—N2—C13	105.43 (10)	O1—C1—C2—C3	−179.39 (12)
N2—Cd1—O3—C20	−127.64 (8)	O1—C1—C6—C5	−179.95 (11)
N1—Cd1—N2—C10	44.42 (8)	O1—C1—C6—C7	2.7 (3)
N1—Cd1—N2—C13	−131.36 (10)	C2—C1—C6—C5	0.03 (19)
N2—Cd1—N1—C7	137.03 (10)	C2—C1—C6—C7	−177.28 (12)
N2—Cd1—N1—C8	−41.86 (8)	C6—C1—C2—C3	0.6 (2)
O1—Cd2—O2—Cd1	28.21 (4)	C1—C2—C3—C4	−0.6 (2)
O1—Cd2—O2—C19	−130.97 (11)	C2—C3—C4—Cl1	179.67 (12)
O2—Cd2—O1—Cd1	−28.38 (4)	C2—C3—C4—C5	−0.2 (2)
O2—Cd2—O1—C1	133.23 (10)	Cl1—C4—C5—C6	−179.00 (9)
O1—Cd2—O2^i^—Cd1^i^	151.79 (4)	C3—C4—C5—C6	0.8 (2)
O1—Cd2—O2^i^—C19^i^	−49.03 (11)	C4—C5—C6—C1	−0.8 (2)
O2^i^—Cd2—O1—Cd1	151.62 (4)	C4—C5—C6—C7	176.87 (12)
O2^i^—Cd2—O1—C1	−46.77 (10)	C1—C6—C7—N1	1.8 (3)
O1—Cd2—O4—C20	−54.67 (16)	C5—C6—C7—N1	−175.58 (13)
O4—Cd2—O1—Cd1	57.15 (5)	N1—C8—C9—C10	−66.99 (16)
O4—Cd2—O1—C1	−141.24 (9)	N1—C8—C9—C11	57.51 (16)
O1—Cd2—O4^i^—C20^i^	−125.33 (16)	N1—C8—C9—C12	177.18 (11)
O4^i^—Cd2—O1—Cd1	−122.85 (5)	C8—C9—C10—N2	70.18 (17)
O4^i^—Cd2—O1—C1	38.76 (9)	C11—C9—C10—N2	−54.36 (17)
O1^i^—Cd2—O2—Cd1	−151.79 (4)	C12—C9—C10—N2	−174.44 (14)
O1^i^—Cd2—O2—C19	49.03 (11)	N2—C13—C14—C15	176.27 (16)
O2—Cd2—O1^i^—Cd1^i^	−151.62 (4)	N2—C13—C14—C19	−3.8 (3)
O2—Cd2—O1^i^—C1^i^	46.77 (10)	C13—C14—C15—C16	−179.71 (14)
O1^i^—Cd2—O2^i^—Cd1^i^	−28.21 (4)	C13—C14—C19—O2	−1.1 (3)
O1^i^—Cd2—O2^i^—C19^i^	130.97 (11)	C13—C14—C19—C18	−179.75 (14)
O2^i^—Cd2—O1^i^—Cd1^i^	28.38 (4)	C15—C14—C19—O2	178.88 (14)
O2^i^—Cd2—O1^i^—C1^i^	−133.23 (10)	C15—C14—C19—C18	0.2 (2)
O1^i^—Cd2—O4—C20	125.33 (16)	C19—C14—C15—C16	0.3 (3)
O4—Cd2—O1^i^—Cd1^i^	122.85 (5)	C14—C15—C16—Cl2	177.90 (13)
O4—Cd2—O1^i^—C1^i^	−38.76 (9)	C14—C15—C16—C17	−0.9 (3)
O1^i^—Cd2—O4^i^—C20^i^	54.67 (16)	Cl2—C16—C17—C18	−177.94 (10)
O4^i^—Cd2—O1^i^—Cd1^i^	−57.15 (5)	C15—C16—C17—C18	0.8 (3)
O4^i^—Cd2—O1^i^—C1^i^	141.24 (9)	C16—C17—C18—C19	−0.3 (3)
O2—Cd2—O4—C20	23.01 (16)	C17—C18—C19—O2	−178.95 (14)
O4—Cd2—O2—Cd1	−58.49 (5)	C17—C18—C19—C14	−0.2 (3)

Symmetry code: (i) −*x*+1, −*y*+2, −*z*+1.

### Hydrogen-bond geometry (Å, º)

**Table:**

*D*—H···*A*	*D*—H	H···*A*	*D*···*A*	*D*—H···*A*
C15—H15···O3^ii^	0.95	2.54	3.248 (2)	131

Symmetry code: (ii) −*x*+1/2, *y*+1/2, *z*.
